# Regarding Bioanalysis Lasting a Few Minutes: Automated Cooling-SPME and Fast-GC for Urinary 2-Phenyl-2-Propanol Monitoring

**DOI:** 10.3390/toxics12100743

**Published:** 2024-10-13

**Authors:** Stefano Dugheri, Niccolò Fanfani, Giovanni Cappelli, Antonio Marigliano, Elisabetta Bucaletti, Donato Squillaci, Ilaria Rapi, Lorenzo Venturini, Giulia Pizzella, Sara Manetta, Alfonso Pavone, Michele Secchi, Iacopo Rainaldi, Nicola Mucci

**Affiliations:** 1 Department of Experimental and Clinical Medicine, University of Florence, Largo Brambilla 3, 50121 Florence, Italy; 2 Eni Health, Safety, Environment & Quality EE, Via Ribotta 51, 00144 Rome, Italy; 3 Eni Energy Evolution, REVT Livorno Refinery, Via Aurelia 7, 57017 Collesalvetti, Italy

**Keywords:** SPME, GC–MS, cooling SPME, full automation, derivatization, cumene, 2-phenyl-2-propanol

## Abstract

An innovative SPME head space GC–MS method, in cooling mode, using a fully automated routine, was developed to detect 2-phenyl-2-propanol, a representative urinary metabolite of cumene. Following an acid hydrolysis and derivatization step with lowered quantities of reagents, acetic anhydride and pyridine, a 30 μm polydimethylsiloxane SPME fiber was used to sample derivatized 2-phenyl-2-propanol, such as benzenemethanol,α,α-dimethyl-acetate, from the headspace. Performances of the method, optimized through experimental design, provide an LOD of 0.034 mg/L and an LOQ 0.10 mg/L, with a short sampling time necessary per sample. The method, developed on standard solutions, will be applied to both occupationally exposed and non-exposed populations.

## 1. Introduction

Cumene, i.e., isopropyl benzene, is a volatile, colorless hydrocarbon and a natural constituent of petroleum. It is present in fossil fuels, solvents, cigarette smoke, and is naturally occurring in the environment in plants, marsh grasses, and foodstuffs [1]. In the workplace, exposure to cumene mainly results from its production and use in the chemical industry, as an intermediate in the production of chemicals such as phenol and acetone, and in styrene, α-methylstyrene, and acetophenone [1]. Cumene classification in the European Union (EU) has been recently updated. According to the Commission Delegated Regulation (EU) 2022/692, amending Regulation (EC) No. 1272/2008 (18th ATP) for its adaptation to technical and scientific progress, it is currently classified as H226 (Flammable Liq 3), H350 (carc 1B), H304 (asp Tox 1) H335 (STOS 3), and H411 (Aquatic Chronic 2) [2]. Although cumene is classified in the EU as a carcinogen class 1B (possible human carcinogen) [3], the occupational exposure limit value is still included in a Chemical Agents Directive (Commission Directive (EU) 2019/1831) [4], establishing a fifth list of indicative occupational exposure limit values, pursuant to Council Directive 98/24/EC and amending Commission Directive 2000/39/EC. Occupational exposure limits, indicated by various regulation organisms, range from 5 ppm to 250 mg/m^3^ in terms of the short time exposure limit (STEL), long time exposure limit (LTE), Threshold Limit Value–Time-Weighted Average (TLV-TWA), and Recommended Exposure Limit (REL) [5,6]. Moreover, the carcinogen classification is A3 (Confirmed Animal Carcinogen with Unknown Relevance to Humans) [7]. Cumene metabolism in the human body leads to the formation of three different products, i.e., 2-phenyl-2-propanol (2P2P), 2-phenyl-1-propanol, and 2-phenylpropionic acid. Among these, around 40% of inhaled cumene is excreted as 2P2P, making it the best choice for biological monitoring, having the highest elimination rate [8]. The Scientific Committee on Occupational Exposure Limits for Chemical Agents (SCOEL) has defined the Biological Limit Values (BLVs) for cumene, sampled within two hours post-shift [9], as 7 mg/g creatinine for 2P2P. Furthermore, cumene has been categorized as a carcinogen, group D (non-genotoxic carcinogen), and notation: skin. A biomarker of exposure has also been identified by the Deutsche Forschungsgemeinschaft (DFG, German Research Foundation), which has identified for 2P2P a Biological Agent Tolerance Value (BAT) of 10 mg/g urinary creatinine [10].

Given the evolving framework of the exposure assessment of cumene, its industrial use, and diffusion, an efficient monitoring method for 2P2P for the potentially exposed population is needed to improve the understanding of its impact on workers.

In previous studies [8,11], the use of solvents, exploiting liquid–liquid extraction (LLE) and gas chromatographic analysis, have been reported to extract the analyte from urine, eliminating most of the interfering compounds. Yet, these procedures result in many manual operations, uncertainty regarding the determination, a higher overall cost, and possible analyte loss.

Generally, a valuable alternative for the determination of 2P2P consists of a reaction with acetic anhydride, often performed in the presence of pyridine, proceeding nearly quantitatively. This reaction represents one of the most common pathways to introduce an acetyl group, which is often employed to protect the hydroxyl moiety [12,13]; moreover, the acetyl-derivative, i.e., benzene methanol,α,α-dimethyl-acetate (BMDA), proves to be feasible for gas chromatography (GC) determination [14,15,16,17]. Despite BMDA embodying proper chromatographic features, methods more and more compliant with the principles of Green Analytical Chemistry (GAC) should be surveyed, regarding the necessity to reduce the use of toxic reagents when possible, and the use of miniaturized techniques in automated workflows. In the last decade, the miniaturization of chromatographic systems has been increasingly implemented, saving both costs and time by automating the sampling procedures; new techniques can simultaneously perform sample collection, extraction, concentration, and injection [18]. In this scenario, Solid Phase Microextraction (SPME), patented by Pawliszyn in 1989 [19], is a solvent-free miniaturized technique that is not exhaustive and enables the combination of sampling, isolation, and enrichment in one step in a fully automated mode, making it suitable for GAC [20,21]. To enable automatic identification, increase stability, and fully automate SPME management, Fast Fit Assemblies (FFAs)-SPME fibers were developed and proposed in 2009 by Chromline (Prato, Italy) [22]. In addition, the use of microbore columns (0.10–0.20 mm inner diameter) enables the analytical throughput to be improved, reducing both the cost and time of analysis for each sample thanks to the lower operating flow of carrier gas required to achieve linear column operating velocities [23]. Among the commercially available coatings, polydimethylsiloxane (PDMS) absorptive liquid coatings can be selected for sampling complex matrices such as urine due to the lack of interanalyte competition [24]. In HeadSpace (HS) SPME, heating the sample solution is advantageous because it increases the HS concentration of volatile compounds [25]; but increasing the sampling temperature decreases the amount of compound captured in the SPME fiber coating because of the exothermic nature of the absorption process. Furthermore, the time required for equilibration between HS and the fiber coating is not negligible and is one of the main drawbacks of SPME sampling. In this context, the cooling of SPME, that is, the heating of the sample and at the same time the cooling of the sample holder, is designed to efficiently extract the analyte into the HS and simultaneously improve the sensitivity of the SPME; it is very efficient in complex matrices where the analyte is strongly adsorbed to the active sites of the sample media [26,27,28,29]. Cooling SPME systems can be classified as (i) internal cooling with liquid CO_2_ directly on the fiber, (ii) internal cooling based on a thermoelectric cooler, or (iii) external cooling using circulating liquids (alcohol or cold water), where the HS of the sample vial is externally cooled [30,31,32,33,34,35,36]. Configuration (iii) was chosen for this work.

We propose a novel fully automated HS-cooling SPME analytical method, after an on-sample derivatization by acetic anhydride in the presence of pyridine, determined by GC and mass spectrometry (MS). The method is optimized throughout the design of experiment (DoE) to reduce the number of experiments needed in the development step and thus to adhere to the GAC principles [37]. The environmental friendliness of the herein proposed analytical method for the quantification of BMDA is evaluated by applying the Green Analytical Procedure Index (GAPI) tool [38]. The performances obtained are compared with the existing analytical procedures to establish a powerful tool for biological monitoring of potentially exposed populations.

## 2. Materials and Methods

### 2.1. Chemical and Reagents

Hydrochloric acid (HCl) 37% (CAS 7647-01-0), acetone (CAS 67-64-1), sodium hydroxide (NaOH) (CAS 1310-73-2), pyridine (CAS 110-86-1), acetic anhydride (CAS 108-24-7), 2-phenyl-2-propanol (CAS 617-94-7), and 4-fluoro phenethyl alcohol (4-FPA) (CAS 7589-27-7) were purchased from Sigma-Aldrich (Saint Louis, MO, USA). Benzenemethanol,α,α-dimethyl-acetate (CAS 3425-72-7) was purchased from Giotto Biotech (Sesto F.no, Italy). The 7, 30, and 100 μm PDMS FFA-SPME fibers were purchased from Chromline (Prato, Italy). Helium (99.999%) was obtained from Air Liquid (Paris, France); HS screw-top 20 mL glass Vials (HSV) (Part No: 5188-2753) and Hdsp cap 18 mm magnetic PTFE/Sil (Part No.: 5188-2759) were purchased from Agilent Technologies (Santa Clara, CA, USA).

### 2.2. Sample Preparation by Fully Automated Procedure

The sample preparation was carried out via a robotic autosampler, following the method developed by Knecht [8]. Each sample (2 mL of urine, prepared as described in the Calibration Levels section) was placed in a 20 mL HSV, where 150 μL of 4-FPA (internal standard (IS), 200 mg/L, in acetone:water 1:1), and 200 μL of HCl 37% were introduced and hydrolyzed for one hour at 90 °C. The temperature of the vials was then lowered to room level, each vial was spiked with 400 μL of a NaOH water solution (12 M) to re-equilibrate the pH, stirred, and then 100 μL of pyridine and 50 μL of acetic anhydride added. The derivatization step was performed at 75 °C for 50 min, followed by cooling assisted SPME absorption at 10 °C for 5 min in HS mode; both operations were performed under stirring. The SPME fiber was then desorbed in the injector for 2 min.

### 2.3. Online Robotic System

The fully automated analytical procedure was developed using a CTC PAL3 RTC System xyz Autosampler (CTC Analytics AG Industrie Strasse 20 CH-4222, Zwingen, Switzerland) equipped with a 1600 mm bar provided with Chronos software ver. 3.5 advance (Axel Semrau GmbH, Sprockhövel, Germany). The apparatus was equipped with a Multi Fiber eXchange (MFX) system and an SPME dual layer extraction (SDLE) developed by Chromline (Prato, Italy), two Pipette Tool (1000 and 200 μL, respectively), a Liquid Syringe Tool (employed to assess the absolute quantity of BMDA; see Results and Discussion section), a Barcode Reader, a Decapper Module, an SPME Fiber conditioning system, and a 20 and a 2 mL tray (CTC Analytics AG, Zwingen, Switzerland). Said configuration was adopted to guarantee an automated routine between reagent dispensation, the shift from syringe to FFA-SPME fiber, the exchange of FFA-SPME fibers, and the cooling SPME sampling.

In order to avoid exposure of chemicals for the operator, due to decapping vials during reagent distribution, the gas chromatographic system was implemented with a dedicated exhaust fumes hood.

The SPME dual layer extraction was equipped with a chiller developed by Fulltech Instruments (Roma, Italy).

**Figure 1:**
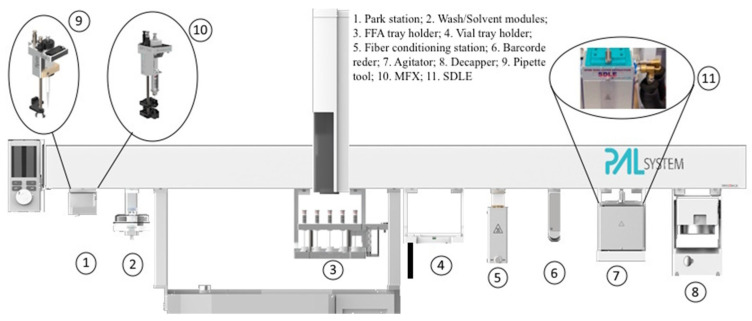
Autosampler for the fully automated determination of 2P2P.

A picture of the autosampler is reported in Figure 1.

### 2.4. Calibration Levels

The working solution of 2P2P was prepared daily in acetone by diluting the stock solution up to a concentration of 20 mg/L. Five calibration levels were prepared by adding proper amounts of said solution to cumene-free urine (Surine^TM^ negative urine control, Merck, Darmstadt, Germany) up to a volume of 2 mL. The concentrations obtained are 0.3, 0.5, 1, 5, and 10 mg/L, respectively. Each standard was then spiked with the internal standard solution and processed as described above.

The absolute quantity of BMDA was calculated on a regression curve obtained by direct liquid injections (1 μL) of toluene solutions in the GC system (5, 10, 50, 100, and 200 mg/L, respectively) to assess the recovery of the method.

### 2.5. GC–MS Operating Conditions

The GC–MS method was developed with a Shimadzu GC-2030 QP2020NX system, with a single quadrupole as the detector.

The column was a Rxi-5Sil MS (10 m, 0.10 mm i.d., and 0.1 μm film thickness) (Restek Corporation, Centre County, PA, USA), and the OPTIC-4 injector port (GL Sciences, Tokyo, Japan) was provided with a 0.75 mm internal diameter liner. The oven settings were isotherm of 35 °C for 2 min, followed by a linear temperature ramp of 20 °C/min to 130 °C, and a subsequent linear ramp of 60 °C/min to 320 °C, held for 2 min. Helium was used as the carrier gas, set at a flow rate of 0.41 mL/min. When operating in cooling SPME mode, the SDLE module, installed on the PAL 6-position agitator, was set at 10 °C using Chronos software.

The mass spectra were initially registered in scan mode (45–200 *m*/*z*; EI energy 70 eV). Then, the acquisition was optimized in SIM mode: ions 136 and 121 were selected as qualifier and quantifier for BMDA, while ions 122 and 109 were selected as qualifier and quantifier for derivatized IS and 4-fluorophenethyl alcohol acetate (4-FPAA), respectively.

### 2.6. Heat Transfer Theory and LTPRI

The theory behind the use of liquid phase SPME as an absorption medium for bioanalysis has been previously described. Louch, Motlagh, and Pawliszyn [39] reported that the extraction time and the square of the coating thickness are inversely proportional, while Wardencki et al. [40] showed that an increase in PDMS thickness enhances the analytes’ recovery. Since the weight of BMDA is 178 Da, 30 μm PDMS was determined as the most suitable sorbent phase. The feasibility of HS-SPME technique for our aim was investigated using the model described in a previous work [41], involving a three-phase system: a liquid polymeric coating, a headspace, and an aqueous solution. The theoretical mass (*n*) of analyte absorbed by a coating after the equilibrium has been reached can be predicted by means of Equation (1), which relates n to the overall equilibrium in a three-phase system:

(1) $$n=\frac{C_{0}V_{1}V_{2}K_{1}K_{2}}{\left(K_{1}K_{2}V_{1}+K_{2}V_{3}+V_{2}\right)}$$

where *K*_1_ represents the partition coefficient between SPME coating and HS, *K*_2_ represents the partition coefficient between HS and the aqueous matrix, *C*_0_ is the aqueous initial concentration of the analyte, and *V*_1_, *V*_2_, and *V*_3_ are the volumes of the coating, the aqueous solution, and the HS, respectively. The partition coefficient between the SPME liquid polymeric coating and the sample, i.e., *K* (defined as *K*_1_·*K*_2_), can be estimated by means of *K*_ow_ [42]. Furthermore, *K*_2_ can be calculated via Equation (2):

(2) $$K_{2}=\frac{K_{H}}{RT}$$

where *K_H_* is Henry’s constant (mol/(atm m^3^)), *R* is the universal gas constant (m^3^ atm/(K mol)), and *T* is the sampling temperature (*K*, in Kelvin scale).

The linear relationship between the solute activity coefficient and Linear Temperature-Programmed Retention Index (*LTPRI*), as shown in Equation (3), can be used further to estimate the *K*_1_ values [43]:

(3) $${\log K}_{1}=\frac{a}{T}+b$$

where *a* is defined as Δ*H_v_*/(2.303*R)* and *b* is defined as [*log*(*RT*/(*γP_vap_*)) − Δ*H_v_*/(2.303RT*)]; Δ*H_v_* (J/mol) is the analyte heat of vaporization, R (8.314 J/(mol K) is the gas constant, *γ* is the solute activity coefficient, *P_vap_* (Pa) represents the vapor pressure, and T* represents the known temperature of coefficient [25].

Establishing a *K*_1_ value with Equation (3) can be laborious and time-consuming. Therefore, a simple yet accurate and reproducible approach to estimating *K*_1_ is based on the *LTPRI* system, as reported in Equation (4):

(4) $$LTPRI=100\times \frac{T_{r}\left(A\right)-T_{r}\left(c\right)}{T_{r}\left(c+1\right)-T_{r}(c)}+100\times c$$

where *T_R_*(*A*) is the analyte retention time, *T_R_*(*c*) is the retention time of the *n*-alkane eluting immediately before the analyte, *T_R_*(*c* + 1) is the retention time of the *n*-alkane eluting immediately after the analyte, and *c* is the number of carbon atoms for *T_R_*(*c*); *LTPRI* values are measured experimentally by GC using the same column employed for BMDA. As shown below, Equation (5) provides a correlation between *K*_1_ and *LTPRI*:

(5) $$log\;K_{1}=0.0042\times LTPRI-0.188$$

### 2.7. Experimental Design Data Analysis

Data were collected using Microsoft Excel and processed using Chemometric Agile Tool (CAT) ver 1.0, an open-source and R-based software [44]. A D-Optimal design was applied: 2 factors, the thickness of the PDMS SPME fiber and the setting in which the sampling was performed, were studied at three levels (7, 30, and 100 µm) and at two levels (sampling performed at 60 °C and at 10 °C), respectively. In Table 1 are reported the experiments performed for the optimization of the method. The following responses were studied: the peak area, the signal-to-noise (S/N) signal, relative standard deviation (RSD), and limit of quantification (LOQ) of the BMDA.

Each experiment was performed in quintuple, and the models were computed at the lower level of quantification, which is 0.10 mg/L; the LOQ was calculated according to the approach based on the standard deviation of the intercept of the linear regression. The aim of this study was to maximize the sensitivity of the analyte and also minimize the other responses.

### 2.8. Greenness Method Evaluation

The Green Analytical Procedure Index (GAPI) was used to evaluate the greenness of the method developed [38]. In this approach, each pentagram of the pictogram represents a step of the analytical protocol, and the color scale, from red to green, suggests high to low environmental impact (figure in Section 3.3).

### 2.9. Method Performance Evaluation

Two control solutions were sampled at 0.3 and 1 mg/L using SPME after derivatization for precision and accuracy tests. The inter-day performance of the method was evaluated on six different days using three different sets of calibration and standard solutions; average curves were constructed each day. Six different sets of calibration and standard solutions, prepared and analyzed sequentially, were used to monitor intra-day performance. The peak area of the analyte, corrected for the internal standard, was plotted against the nominal concentration of each calibration solution to obtain the 2P2P calibration curve. Least squares linear regression was used to obtain the best-fitting function. Reliable limit of detection (LOD) and limit of quantitation (LOQ) values were obtained using the standard deviation (SD) of the response and the slope approach; in fact, LOD values were strongly influenced by both the stability of the background noise and the reproducibility when an S/N evaluation was applied. Therefore, the estimated SDs of the responses were calculated from the standard deviations of the Y-intercepts (SDY-Is) of the regression curves. Precision was assessed by the RSD of the quantitative data derived from the replicate analysis of the control solutions, while accuracy was determined by calculating the yield between the determined and nominal amounts. The concentration range for the calibration curves was the same as described in the Calibration Levels section.

## 3. Results and Discussion

The evolving framework of the European and national legislation on protection from dangerous substances has emphasized the need for a deeper understanding of human exposure to chemicals. Biological monitoring is one of the methods by which it is possible to achieve this goal. Even though cumene exposure has been studied in the past, mainly because of its use in the chemical industry [45], its new classification requires a greater and more detailed in-depth analysis, especially in the workplace where a potential exposure, even if at a low level, is possible. The development of analytical methods, aimed at ensuring workers’ health protection, is therefore a key element in this context [21].

**Figure 2:**
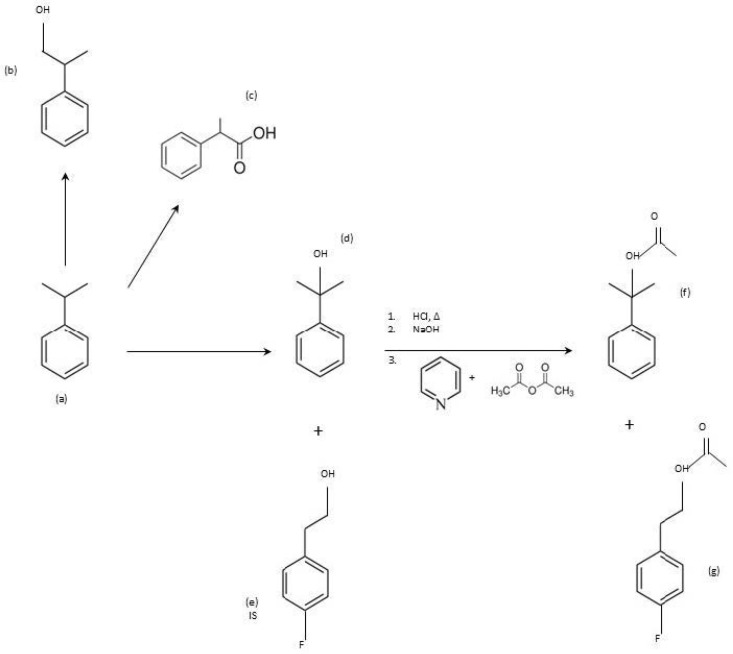
Metabolites generated from cumene (**a**) exposure: 2-phenyl-1-propanol (**b**), 2-phenylpropionic acid (**c**), and 2-phenyl-2-propanol (**d**). Hydrolysis of 2P2P and 4-FPA ((**e**), IS) and subsequent derivatization with acetic anhydride in presence of pyridine, leading to the formation of BMDA (**f**) and 4-FPAA (IS derivative, (**g**)).

The derivatization using acetic anhydride in aqueous matrices and subsequent SPME sampling was first introduced by Pawliszyn for the derivatization of phenols [46]; this method has been applied to a large variety of compounds containing hydroxyl groups, mainly phenols, in both the on-sample and on-fiber modes [47,48,49,50]. Nonetheless, to our knowledge, this is the first work where a fully automated cooling SPME routine is combined with an aqueous derivatization with acetic anhydride. The CTC PAL3 RTC System xyz custom autosampler, installed online with the GC, improves productivity, minimizes the dead time between samples, and reduces the cost of the analytical test; the full automation of the system requires minimal operator supervision and enables more samples to be processed during each analytical run. A brief scheme of the metabolites generated after cumene exposure and the subsequent derivatization of the selected analyte (in the presence of the IS) are reported in Figure 2.

### 3.1. SPME Heat Transfer Theory and LTPRI Calculation

Since SPME performs extraction at the equilibrium stages, it is not exhaustive; therefore, the hypothesis of the ideal conditions required by the mathematical modeling must be verified. The estimation of the distribution constants from the physico-chemical tables or by the structural unit contribution method can anticipate the trends in the SPME analysis. To calculate the theoretical mass, using Equations (1) and (2), we considered the concentration of the middle calibration level employed in the method presented, i.e., 0.3 mg/L (normalized for the final volume and converted in BMDA), with *V*_2_ and *V*_3_ at 2.9 mL and 17.1 mL, respectively. The physico-chemical constants of the BMDA were obtained from Performs Automated Reasoning in Chemistry (SPARChem, Danielsville, GA, USA). The calculation provided theoretical *n* values of 120 ng (at equilibrium); conversely, the experimental mass loaded on the fiber amounted to around 99 ng (also determined at the equilibrium stage). The sampling time working at 60 °C was established at 15 min despite the equilibrium being reached around 38 min; in this condition, the mass uptaken was 21 ng.

Furthermore, for HS-SPME, when the sampling temperature is different from the temperature at which *K_1_* was established, i.e., 10 °C versus 60 °C, the effects on the partition coefficients can be predicted [48]. In particular, the linear relationship between *K*_1_ and temperature can be used to correct the *K*_1_ value with an experimental approach, unlike Equations (1) and (2) presented above [43].

As for the evaluated *LTPRI* and *K*_1_ values (1384 and 4.23 × 10^5^, respectively) of BMDA, the fact that this theoretical approach could facilitate using SPME for compound qualification is reinforced [25,51,52], indicating also an improvement in absorption capacity when the SPME fiber is operating in cooling mode (10 °C). In fact, the theoretical mass of the absorbed analyte increased to 148 ng, with an experimental value of 167 ng (at equilibrium equal to 14 min). This result provides important consequences: above all, the possibility of employing a reduced sampling time, and therefore an improvement in the analytical throughput, enhancing the number of samples processed and reducing the costs.

The sampling time was then set to 5 min when operating in cooling SPME mode. In this condition, the experimental mass loaded was 53 ng. Table 2 reports the physico-chemical parameters obtained for BMDA, together with the time required to reach equilibrium (experimentally determined), and the theoretical and experimental mass values uptaken by the SPME fiber at equilibrium for both operating temperatures (60 °C and 10 °C, respectively) and both absorption times (15 and 5 min, respectively).

### 3.2. Experimental Design

A D-Optimal design was adopted to investigate the possible interactions among the selected variables involved in the SPME sampling step, performing only 5 + 1 experiments; the sixth experiment was performed only to validate the models. As reported in Table 1, the thickness of the SPME fiber was studied at three levels: 7, 30, and 100 µm, while the SPME sampling mode was studied at two levels, with the vial heated at 60 °C and at 10 °C. The model describing the S/N signal (Y_2_) of BMDA was not statistically significant, and, thus, it is not reported. Conversely, the models concerning the sensitivity of the analyte (Y_1_), LOQ (Y_3_), and RSD (Y_4_) were validated at the test point since the predicted responses were not significantly different from the experimental ones (confidence interval 90%). Evident in Figure S1 of the Supplementary Materials, the experimental conditions enabling the highest sensitivity for the analyte and the lowest LOQ and RSD were obtained working with the 30 µm fiber in the cooling SPME setting for the sampling step.

It is evident from the results (see the response surfaces in Figure S2 of the Supplementary Materials) that the models describing the sensitivity and thus the LOQ were mainly affected by the temperature at which the sampling was performed, while the fiber thickness has a minor effect on the formation of the metabolite. On the other hand, the model describing the RSD was affected in equal measure by the two factors, and the parameter was minimized by working with x_1_ = 0 and x_2_ = 1.

The analysis of the model for the S/N signal (Y_2_) demonstrated a similar performance to the one of the peak area (Y_1_), even if it was not validated at the test point, suggesting that the experimental conditions with x_1_ = 0 and x_2_ = 1 were also suitable to maximize this parameter.

### 3.3. Green Analytical Protocol Index (GAPI) and Method Comparison

**Figure 3:**
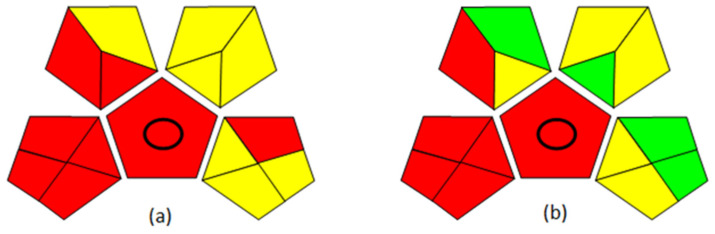
(**a**) GAPI pictogram of the method described by Knecht; (**b**) GAPI pictogram of the method developed in this paper.

The evaluation of the green character of an analytical protocol has gained great importance: in particular, reduced use of toxic reagents by the miniaturization of the procedures as well as reduced exposure for the operator are the main key points we addressed. For this reason, the greenness of the analytical method developed in this work for the determination of the urinary 2P2P was compared to that reported by Knecht [8]. In the literature, we can find different tools and software able to assess the sustainability of a method, such as the National Environmental Methods Index (NEMI) [53], the Analytical Eco-Scale [54], and the Analytical Method Greenness Score (AMGS) calculator [55]. To the authors’ knowledge, the GAPI was the one that best fit the purpose [38]. Figure 3a,b describes the method reported by Knecht [8], while Figure 3b describes the method we proposed. As shown, the first pentagram on the bottom left, referring to the sample collection and storage, has an equal impact on the sustainability of the methods since it is the same for both procedures. Conversely, the pentagrams on the upper left and upper right are quite different: in one case (Figure 3a), the sample pre-treatment involves a step of macroextraction (LLE) with large amounts of reagents used, while, in the other case (Figure 3b), the sample pre-treatment involves a microextraction with a greener character. Lastly, the pentagram on the bottom right differs for the two methods regarding the quantity of generated waste and the hazard for the operator as the energy required for the acquisition of the data can be considered equal for the two methods.

Thus, performing the derivatization directly on the aqueous matrix, together with the use of an SPME fiber to sample the analyte, instead of an LLE procedure, is safer for the operators; moreover, it represents a lower environmental impact in terms of the solvents and reagents utilized, appropriately fitting the aim of compliance with the GAC principles.

Moreover, acetic anhydride was recently classified in between “recommended” and “problematic” as far as greenness goes, suggesting a tolerance in its use in small amounts [56]; in our opinion, 50 µL for each sample, with a related limited cost (around EUR 0.1 for 20 reaction cycles) represents a good compromise. The future implementation of the method will surely need to address this aspect, as well as the use of pyridine, classified in between “problematic” and “hazardous” [56]. Nonetheless, its reduced use, i.e., 100 µL per sample, can be tolerated despite the lack of compliance with the GAC principles.

The use of MS compared to a Flame Ionization Detector (FID) [8] enables the easier attribution of the peaks observed in the chromatogram, safeguarding against wrong identifications and assignments.

Hence, from said comparison, we believe that the proposed procedure represents a balanced compromise between high sensitivities and compliance with the GAC principles, as can be easily visualized from the color scale of the pictograms.

### 3.4. Method Performances

**Figure 4:**
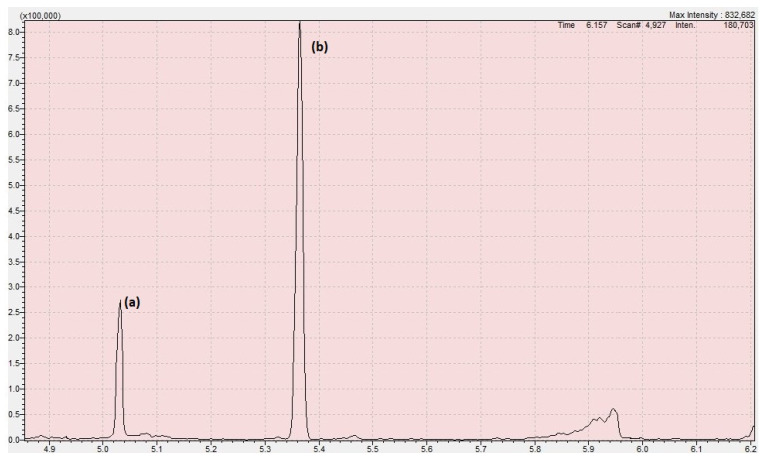
Chromatogram obtained in our experimental conditions, displaying (**a**) BMDA and (**b**) 4-FPAA, for the 0.5 mg/L calibration level.

**Figure 5:**
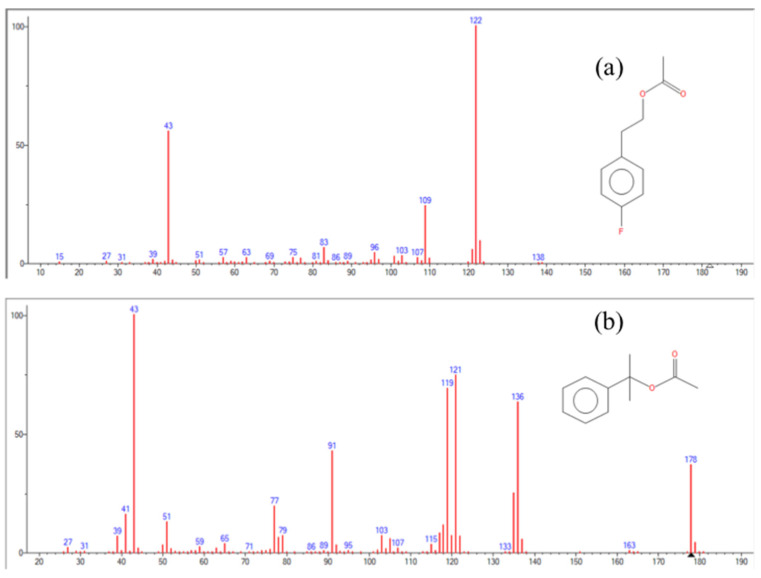
(**a**) Mass spectra of 4-FPAA; (**b**) BMDA.

The chromatogram obtained with the developed method is shown in Figure 4, the relative mass spectra are shown in Figure 5, and the parameters of the calibration curve obtained are shown in Table 3. In these experimental conditions, BMDA showed a retention time of 5.03 min, while 4-FPAA showed a retention time of 5.36 min. A high throughput was therefore obtained, with total control regarding the whole process thanks to the full automation of the procedure, i.e., 31 samples processed per day, considering a total time of 17 min from the SPME sampling to the end of the chromatographic run, while maintaining a high sensitivity. In this way, the pre-analytical errors were minimized, as well as the chemical exposure of the operator.

As detailed in the Materials and Methods Section 2.9, the method’s performance was evaluated as follows: the daily LOD was calculated by multiplying 3.3 by the ratio between the standard deviation of the Y-intercept and the slope of the average daily curve, and the LOQ is three times the LOD according to ICH guidelines [57]. The RSD% for each day was calculated as the mean ratio between the standard deviation and the daily average of the control levels multiplied by 100. We evaluated the daily accuracy by calculating the mean of variation coefficients (CVs%), defined as the ratio between the daily average of the experimental values of the control levels (obtained via GC–MS) and the theoretical value (the nominal concentrations of the control levels multiplied by 100). The inter-day accuracy is the mean of the six average daily CVs% of the control levels, each obtained from the three repetitions. The intra-day accuracy is the mean CVs% resulting from six repetitions on the same day of the control levels. The performance evaluations are reported in Table 4: the method shows good linearity (R^2^ > 0.990), while the LOD and LOQ result in values in the sub-ppm range; the data reported include the mean between the inter-day and intra-day outcomes. The developed method shows an improvement in terms of sensitivity compared to Knecht et al. [8], with an LOQ of about an order of magnitude below. In fact, despite the sample pre-treatment and derivatization employed being the same, the combined use of MS and cooling SPME sampling enables investigations at lower concentration scenarios. Future improvements regarding the method will investigate the possibility of introducing modifications, e.g., performing the derivatization with other reagents, to further enhance the sensitivity. Additionally, surveying the substitution of hazardous compounds (e.g., pyridine) to better fit with the GAC principles will need to be addressed as well.

## 4. Conclusions

The monitoring of urinary 2-phenyl-2-propanol, the main biological marker of cumene exposure, requires an effective analytical approach due to an increasing awareness regarding cumene toxicity and the related lowering of the occupational limits, which are becoming more and more strict. In this framework, we developed a novel fully automated GC–MS method including prior optimization with chemometric tools to maximize its sensitivity. The best experimental conditions were found using a 30 µm PDMS SPME fiber to sample the acetyl-derivative, i.e., benzenemethanol,α,α-dimethyl-acetate, including prior derivatization with acetic anhydride in the presence of pyridine, operating in cooling SPME mode with the temperature set to 10 °C. The method developed was tested on standard solutions, providing excellent sensitivity. Future improvements, compliant with green chemistry, will be surveyed, especially in the substitution of hazardous substances (such as pyridine). Concerning its application, further studies will investigate its applicability among both occupationally exposed and non-exposed populations.

## Figures and Tables

**Table 1:** Experimental matrix with the corresponding experimental plan.

	Experimental Matrix		Experimental Plan	
Exp#	X1	X_2_	SPME Fiber Thickness	HS Sampling Temperature
1	−1	−1	7 µm	60 °C
2	0	−1	30 µm	60 °C
3	1	−1	100 µm	60 °C
4	−1	1	7 µm	10 °C
5	0	1	30 µm	10 °C
6	1	1	100 µm	10 °C

**Table 2:** Partition coefficients, theoretical mass loaded, equilibrium time, and experimental mass loaded for BMDA.

	*K*_1_ = *K*_ow_/*K*_2_	Henry’s Constant (atm m^3^/mol)	*K*_2_ (=*K_H_*/*RT*)	*K*_ow_ (log)	t_eq_ (min)	*n* (ng) *C*_0_ = 0.3 mg/L Static HS SPME	*n* (ng) *C*_0_ = 0.3 mg/L	*n* (ng) *C*_0_ = 0.3 mg/L
60 °C	3.95 × 10^4^	5.25 × 10^−4^	1.92 × 10^−2^	2.88	38	120	99 ^a^	21 ^b^
10 °C	4.23 × 10^5^	5.58 × 10^5^	2.40 × 10^−3^	3.41	14	148	167 ^a^	53 ^c^

(R = 8.2054 × 10^−5^ m^3^ atm/(mol K)); ^a^—measured at equilibrium; ^b^—measured at 15 min; ^c^—measured at 5 min.

**Table 3:** Calibration parameters.

R^2^	Slope	Intercept
0.991	210,501	151,459

**Table 4:** Performances of the method.

LOD (mg/L)	LOQ (mg/L)	RSD %		Recovery (%)			Accuracy (%)	R^2^
		0.3 mg/L	1 mg/L	0.3 mg/L	1 mg/L	10 mg/L		
0.034	0.10	4.38	4.12	92.1	95.7	96.0	93.6	0.991

## Data Availability

The original contributions presented in the study are included in the article/Supplementary Materials. Further inquiries can be directed to the corresponding author.

## Supplementary Materials

The following supporting information can be downloaded at https://www.mdpi.com/article/10.3390/toxics12100743/s1, Figure S1: Overlapping of the contour plots obtained computing the models for peak area of BMDA (blue lines), LOQ (green lines), and RSD% (red lines); Figure S2: Response surfaces obtained for the models of BMDA. Y_1_ = peak area; Y_3_ = LOQ; Y_4_ = RSD%.

## Abbreviations

EU: European Union; STEL: Short time exposure limit; LTE: Long time exposure limit; TLV-TWA: Threshold Limit Value–Time-Weighted Average; REL: Recommended Exposure Limit; 2P2P: 2-phenyl-2-propanol; SCOEL: Scientific Committee on Occupational Exposure Limits for Chemicals Agents; BLVs: Biological Limit Values; BATs: Biological Agent Tolerance Values; LLE: Liquid–liquid extraction; BMDA: Benzene methanol,α,α-dimethyl-acetate; GC: Gas chromatography; GAC: Green Analytical Chemistry; SPME: Solid Phase Microextraction; FFAs: Fast Fit Assemblies; PDMS: Polydimethylsiloxane; MS: Mass spectrometry; DoE: Design of experiment; GAPI: Green Analytical Procedure Index; HSV: HS screw-top 20 mL glass Vials; 4-FPA: 4-fluoro phenethyl alcohol; HCl: Hydrochloric acid; NaOH: Sodium hydroxide; IS: Internal standard; MFX: Multi Fiber eXchange; SDLE: SPME dual layer extraction; 4-FPAA: 4-fluorophenethyl alcohol acetate; CAT: Chemometric Agile Tool; RSD: Relative standard deviation; S/N: Signal-to-noise; LOQ: Limit of quantification; LOD: Limit of detection; SDY-I: Standard deviation of Y-intercept; NEMI: National Environmental Methods Index; AMGS: Analytical Method Greenness Score; CVs%: Variation coefficients.
