# Supplementary material for: Regarding Bioanalysis Lasting a Few Minutes: Automated Cooling-SPME and Fast-GC for Urinary 2-Phenyl-2-Propanol Monitoring
Source: Toxics. 2024 Oct 13;12(10):743. doi: 10.3390/toxics12100743 (PMC11511570; doi:10.3390/toxics12100743)
Supplement: Supplementary file 1 [file toxics-12-00743-s001.zip › Toxic submission SM.pdf]

**Supplementary materials**

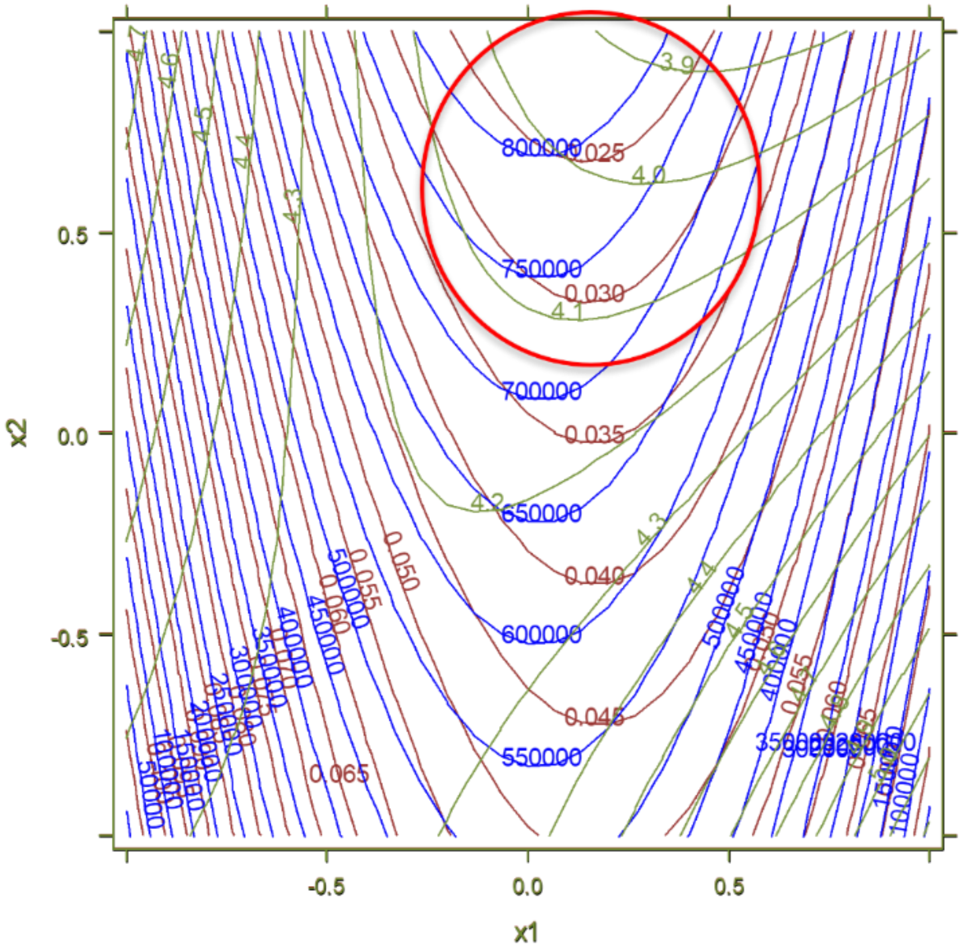


**Figure S1.** Overlapping of the contour plots obtained computing the models for peak area of BMDA (blue lines), LOQ (green lines) and RSD% (red lines).

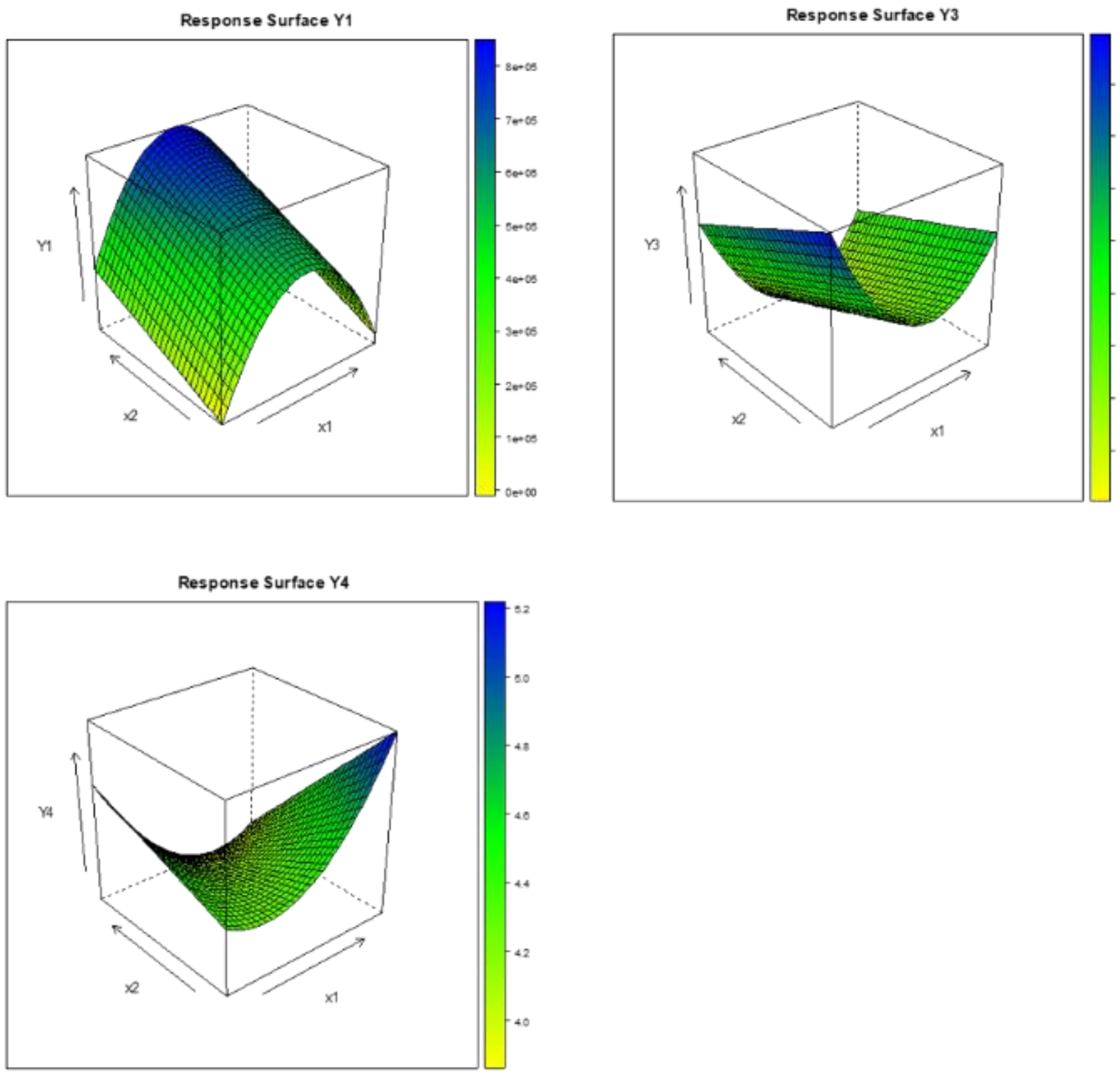


**Figure S2.** Response surfaces obtained for the models of BMDA. $Y_1$ = peak area, $Y_3$ = LOQ, $Y_4$ = RSD%.
